# Feasibility, safety and acceptability of select outcome measures in a physiotherapy study protocol for boys with haemophilia

**DOI:** 10.1186/s40814-021-00831-1

**Published:** 2021-05-06

**Authors:** Nicola Thorpe, Phillip Harniess, Eleanor Main, Nicola Hubert, Sarah Rand, David Stephensen, Ri Liesner, Melanie Bladen

**Affiliations:** 1 Great Ormond Street Hospital for Children NHS Foundation Trust, London, UK; 2 UCL Great Ormond Street Institute of Child Health, London, UK; 3 Kent Haemophilia Centre, Kent and Canterbury Hospital, Canterbury, UK

**Keywords:** Feasibility, iSTEP, Children, Haemophilia, Outcomes, Myometry, 10-m ISWT

## Abstract

**Background:**

There is a lack of functional performance measures for children and young people with haemophilia (CYPwH) with associated control data from typically developing boys (TDB). The literature advocates development of a core set of outcome measures for different chronic conditions. As medical treatment improves, CYPwH are experiencing better outcomes; therefore, more challenging measures are required to monitor physical performance. Such testing is not performed routinely, due to practical and safety concerns.

**Aim:**

Evaluate the feasibility, safety and acceptability of select outcome measures as part of a study protocol testing CYPwH; including myometry, 10 metre incremental shuttle walk test (10-m ISWT), iSTEP (an incremental step test, with data from TDB), and 1 week of accelerometry-wear at home.

**Methods:**

Sixty-six boys aged 6–15 years with mild, moderate or severe haemophilia A or B (including inhibitors) attending routine clinics at Great Ormond Street Hospital were approached to participate. Descriptive statistics and content analysis were used to assess outcomes of feasibility, safety and acceptability, which included recruitment/retention rates, protocol completion within routine appointment timeframes, performance testing without serious adverse events/reactions (SAE/SARs), and acceptability to CYPwH of high-level performance measures.

**Results:**

Outcomes were met: 43 boys completed testing at clinic review (Jan–Nov 2018) within a 10-month timeframe, retention was 95% at completion of protocol and no SAE/SARs were reported throughout testing.

**Conclusion:**

Feasibility, safety and acceptability of the study protocol have been established in this population. Both high-level performance tests, iSTEP and 10-m ISWT, were an acceptable addition to boys’ routine clinic appointments and could be safe, acceptable choices of outcome measure as part of a core set of tests for CYPwH. Further investigation of the psychometric properties for the iSTEP is now justified, in order for it to be used as a standardised, validated, reliable outcome measure in clinical or research settings.

**Trial registration:**

Retrospectively registered on September 3, 2019, on ClinicalTrials.gov (ID: NCT04076306).

**Supplementary Information:**

The online version contains supplementary material available at 10.1186/s40814-021-00831-1.

## Key messages regarding feasibility (see Additional file 1 for the Consort Checklist)

1. What uncertainties existed regarding the feasibility?

- The safety and acceptability of high-level performance testing in children and young people with haemophilia (CYPwH).
- Could data for the select outcome measures be collected in a timely manner whilst CYPwH attend for routine clinic reviews?
- What is the recruitment and retention rate over a 1-year period; indicating whether further research, including a larger clinical intervention trial, could be successful in the future?

2) What are the key feasibility findings?

- Outcome measures implemented in this protocol were safe and acceptable for CYPwH.
- It was feasible to carry out the study protocol at routine clinic review.
- Recruitment and retention rates indicate that CYPwH and their families are invested in participating in the research process.

3) What are the implications of the feasibility findings for the design of the main study?

- Safety and acceptability have been demonstrated, which justifies further research to establish the psychometric properties of the new performance measure, the iSTEP, in this population of CYPwH.

If psychometric properties are established, the iSTEP could be used as a new standardised, validated, reliable outcome measure for CYPwH. With these properties, it could be used to demonstrate change in larger intervention studies or in clinical settings.

## Introduction

Conducting research is of high importance for the physiotherapy profession, in order to extend the growing evidence base for appropriate, efficacious, cost-effective management approaches [1]. In a recent study, the top 10 generic research priorities for UK Physiotherapists were identified and included those of effectiveness and measurement of physiotherapy intervention [1]. This was in collaboration with the James Lind Alliance (JLA); a group which aims to identify the research priorities of patients, carers and clinicians to ensure these are being reflected in the work carried out by researchers. Condition-specific priorities have also been investigated by the JLA and uncertainties for people with haemophilia include, ‘what is the role of exercise for both prevention and treatment of joint damage in haemophilia?’ and ‘are there factors other than the number of joint bleeds that are associated with haemophilic arthropathy (joint damage)?’ [2]. The research described in this article aims to begin addressing these uncertainties.

In addition, the National Institute for Health Research (NIHR) has recently placed an emphasis on feasibility studies [3], which are considered to be good value for money as they gather information to determine whether a full clinical trial would be successful; for example, by demonstrating ability to recruit patients, before a full trial is conducted.

This study encompasses these recommendations by using a feasibility design to investigate safety and acceptability of high-level performance outcome measures not previously used in assessment of children and young people with haemophilia (CYPwH). The aim is to develop the use of outcome measures which help assess performance, guide physiotherapy intervention and are sensitive enough to measure change in this cohort.

Haemophilia is an ‘X’ chromosome-linked genetic disease, almost exclusively occurring in boys, characterised by bleeding into the joints; predominantly the ankles, knees and elbows, due to missing or low levels of blood clotting factor VIII or IX (haemophilia A or B, respectively). Recurrent joint bleeds can lead to joint damage (synovitis or arthropathy) and functional impairment. Bleeding occurs mostly after trauma or injury but can appear to be spontaneous. Disease severity, classed as mild, moderate or severe, correlates with the percentage of normal levels of clotting factor present (mild 5–40%, moderate 1–5% and severe <1%). Some children with haemophilia develop an ‘inhibitor’, whereby they make antibodies, which reduce treatment efficacy. Novel treatments are being developed for this problem. Factor replacement therapy is the mainstay of treatment, given via regular injections to prevent bleeds (prophylaxis); and/or in response to an injury or suspected bleed (on demand). In recent years, treatment has significantly reduced annual bleed rates and improved quality of life for CYPwH, bringing their functional ability more in-line with unaffected peers [4].

However, in terms of functional outcomes, there is still progress to be made; CYPwH have demonstrated reduced fitness and muscle strength that may impact on physical performance [5, 6, 7]. Barriers to exercise exist, including activity restriction due to parental concern, risk or fear of bleeds, musculoskeletal pain or deconditioning, and a lack of disease specific guidelines for ‘safe’ physical activity participation [7, 8]. Reduced physical activity levels have implications for on-going health. Adults with haemophilia are more likely to be sedentary given past recommendations about exercise, limited treatment options and resultant poor joint function. As such, risk factors for cardiovascular disease associated with a sedentary lifestyle, such as obesity and hypertension, are more prevalent in adults with haemophilia than in the general population [9, 10].

Due to ongoing advances in medical treatment, previous haemophilia-related comorbidities are unlikely to persist in the future. Today, CYPwH are experiencing improved health outcomes and can expect treatment to prevent or minimise joint damage and even facilitate a bleed-free life [4]. In a recent UK study, more than half the CYPwH had no obvious haemophilia-related joint damage as measured by the Haemophilia Joint Health Score (HJHS vs 2.1) [11]. The HJHS is currently the main outcome measure routinely used in physical assessment of CYPwH. Whilst the study demonstrated good joint health in those children, it also highlighted how the HJHS now lacks sensitivity in optimally managed cohorts. As treatment for haemophilia improves, so too does the need for enhanced physical performance tests, which are sensitive to sub-clinical joint problems and associated functional or endurance deficits.

Such outcome measures should be portable, easy to administer and simple to perform by CYP. They need to measure higher intensity activity correlating with every-day life of CYP and provide clinically useful information (e.g. to inform exercise prescription) both in the short and long term [8, 12]. There is limited evidence in the literature regarding use of high-level performance measures in CYPwH, but it is recognised that they should be safe and acceptable; with established psychometric properties and associated normal data [6], prior to use as outcome measures in clinical practice or research.

### Study aims

 1. Investigate the feasibility, safety and acceptability of implementing a study protocol, including two high-intensity performance tests plus measures for joint health, lower-limb muscle strength and daily activity levels in CYPwH.

2. Specifically, to evaluate the safety and acceptability of a new performance measure, the iSTEP, in CYPwH.

## Methods

Ethical approval for the study was gained from the Central London Research Ethics Committee (ref: 17/LO/1192). Convenience sampling aimed to recruit at least 40 participants prospectively from forthcoming clinic lists during January–November 2018, from the Great Ormond Street Hospital for Children NHS Foundation Trust Haemophilia Comprehensive Care Centre (GOSH). Thirty is the proposed minimum number of participants for each arm of a feasibility trial [13]. The feasibility study was non-interventional and non-randomised. Boys were identified by clinical staff using inclusion and exclusion criteria (Table 1).

**Table 1:** Inclusion and exclusion criteria

Inclusion criteria	Exclusion criteria
Boys with mild, moderate and severe haemophilia type A or B. Age 6–15 years. Inhibitor or non-inhibitor. Able to follow simple verbal instructions and provide informed consent.	History of: fracture or trauma to the lower limb; orthopaedic surgery of the lower limb; Acquired brain injury; or any other disturbance of the central nervous system. Joint or muscle bleed in the lower limb in the past 6 weeks. Lower limb pain (assessed prior to testing). Unable to follow verbal instructions. Diagnosis of uncontrolled severe asthma, exercise induced bronchoconstriction or other medical conditions.

Outcomes measured were joint health using the HJHSv2.1; lower limb muscle strength using myometry, two performance measures—the 10-m Incremental Shuttle Walk Test (10-m ISWT) and the iSTEP, and daily physical activity patterns using accelerometry-wear for a 1-week period at home, immediately following testing.

**Fig. 1:**
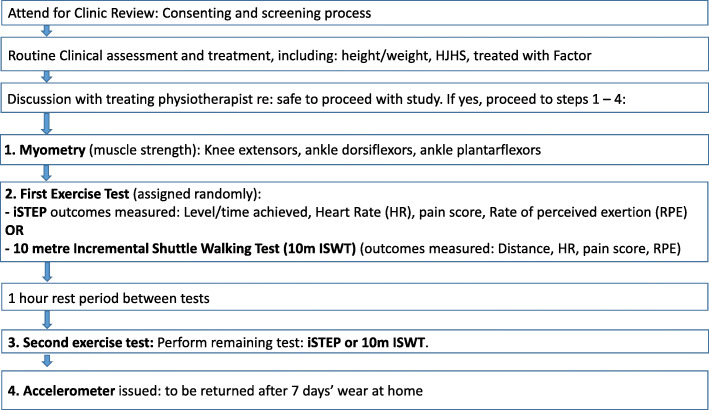
Schedule of testing

Once consented, boys were screened prior to testing, including specific questions about pain or symptoms of a bleed, on the day of testing and in the previous 6 weeks. If so, they were asked to identify presence of pain on a body chart and give a score using the Wong-Baker Faces Pain Rating Scale (WBFPRS). Reports of pain or bleeds were referred to the Specialist Haemophilia Physiotherapy Team who reviewed to confirm inclusion/exclusion. Boys received prophylaxis on the day of testing if this was usual for them. Figure 1 shows schedule of testing.

### Feasibility, safety and acceptability measures

Descriptive statistics and content analysis were used to assess outcomes of feasibility, safety and acceptability of the study protocol.

### Feasibility

The National Institute for Health Research (NIHR) [3] defines the parameters of feasibility studies, which include whether the proposed study can be done, determining recruitment processes and rates, improving study design methods for bigger intervention studies, identifying suitability of outcome measures, adherence/compliance rates, and time required to collect and analyse data. A feasibility metric [14] was adapted, to assess feasibility outcomes for this study and include; process, resource, management and scientific aspects. Table 4 shows this in more detail.

### Safety

This was measured by rate of Serious Adverse Events or Serious Adverse Reactions (SAEs and SARs) described in NIHR Good Clinical Practice (GCP) Guidelines [16] and defined as a joint or muscle bleed occurring in the duration of this study, including the week of activity monitoring.

### Acceptability

Subjective reports about the preferred physical performance measure were noted, from participating boys. Reported pain levels, numbers declining participation and withdrawal rate from the study were also used as indicators of acceptability.

### Patient-centred outcome measures of the study protocol: 25-level 10-m incremental shuttle walking test (10-m ISWT)

The original 10-m ISWT [17] has been modified to increase the number of levels walked or run [18] and is validated in children [19, 20]. The further extended and validated 25-level 10-m ISWT [21] was used in this study. Each participant was allowed to start running at any point in order to keep pace with the beeps. Testing ended when they were unable to go any further or if they did not match pace for 2 consecutive shuttles. Pain, heart rate (HR) and rate of perceived exertion (RPE) were recorded at rest, immediately after and 3 min after-testing. Total distance achieved was documented. Forty-five minutes was allowed to administer, including travel time to designated area.

### iSTEP

The iSTEP is a new and valid submaximal exercise step test [22]. It has associated normal data from unaffected CYP (see Additional file 2). A standard 3-height adjustable exercise step was used for iSTEP testing. Shank length measurement (most prominent points of the head of fibula to lateral malleolus) was halved and rounded down to 15, 20 or 25 cm, determining step height during testing. A practice was given and boys stepped in time to externally paced beats on a standardised audio track, becoming incrementally faster over 5 2-min levels. Stepping was led by the right foot up, left foot up, right foot down and left foot down. The leading leg was swapped at each level. All boys were advised to work as hard as possible to complete the test; stopping at any point, if they were too tired or unable to continue. iSTEP outcomes measured were last completed iSTEP level achieved before stopping (levels 0–5), total duration of stepping (0–10 min), completion achieved or not, pain, RPE and HR taken before, immediately after and 3 min after-testing. 15–20 min was allowed to administer the test.

### Physical activity levels

ActiGraph GT3X bi-axial accelerometers were worn for 7 days, on a velcro waist strap. They were removed at night and for any water-based activities and returned in a stamped addressed envelope, in line with comparable accelerometry studies in boys with haemophilia [15, 23, 24]. An education session regarding use was provided to participants and parents, plus a letter for school explaining study participation and accelerometry-wear. Data from each accelerometer were uploaded using ActiGraph Firmware and standard accelerometry outcomes were calculated, including average daily time spent in sedentary, light, moderate and vigorous activity. Five to 10 min was allowed to explain use and demonstrate wear/positioning.

### Myometry

A hand-held Lafayette manual muscle test system, model 01163 dynamometer was used to obtain measurements for maximum voluntary isometric muscle strength (in Newton-metres, Nm) of the knee extensors, ankle plantarflexors and dorsiflexors, following a standardised protocol [25]. 20 min was allowed to complete.

### Pain

The Wong-Baker Faces Pain Rating Scale (WBFPRS) (wongbakerfaces.org) was used to assess pain during screening; and before, during and after performance testing. Unexpected changes in pain scores may have indicated adverse effects and conversely, stable pain scores add to the overall picture of acceptability and safety of performance testing. Reliability and validity of the WBFPRS have been demonstrated in children 3—18 years old [26]**.** About 1 min was required to explain and rate.

### Rate of perceived exertion (RPE)

RPE was assessed using the Boys’ OMNI stepping scale, which has been validated as a measure of RPE, correlating with oxygen consumption and heart rate in children aged 8–12 years, for a variety of exercise modalities and intensities [27]. It is a self-reported numerical scale of 0–10, with pictures and descriptors from, ‘not tired at all’ to ‘to very, very tired’. About 1 min was required to explain and rate.

### Haemophilia Joint Health Score (HJHS)

The HJHS version 2.1 is a validated measure of the ankle, knee and elbow joint health including range of movement, swelling and strength plus a category for gait performance [28]. Scoring ranges from 0 to 124; lower scores indicating better joint health. HJHS assessment was part of routine clinic review for all CYPwH, except for those with mild disease reporting no problems; for whom it was additional to usual care. Forty-five to 60 min was allowed to administer.

## Results

Data from patient-centred outcome measures included in this study will be analysed and reported in future publications. Feasibility, safety and acceptability of the study protocol will be reported in the following section.

### Recruitment

**Fig. 2:**
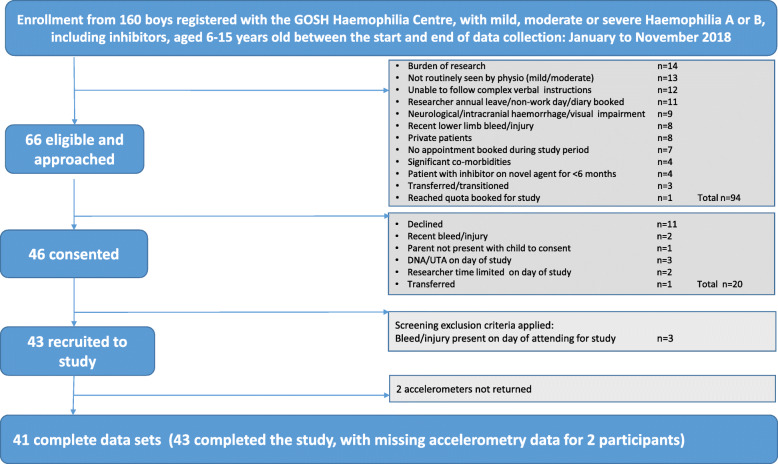
Recruitment to study

Forty-three boys were recruited to the study: 23 were randomly assigned to complete the iSTEP first; 20 to complete the 10-m ISWT first, using a random allocation generator. During the iSTEP, 2 boys were tested at the 20cm step height and the rest at 15cm. No participants withdrew from the study (Fig. 2). Time commitment totalled 1.5–3 h in addition to routine assessment, plus 1 week of accelerometry-wear at home, starting the next day. The varied time commitment of 1.5–3 h was dependent on availability of the participant for testing, during their usual clinic review process. The same tester, NT, carried out all recruitment to the study and testing (with the exception of one iSTEP test, carried out by PH); and all participants completed the same elements of the protocol; therefore, blinding was not implemented.

### Participant characteristics

There were 5 boys with mild, 5 with moderate and 26 with severe haemophilia and 7 boys had inhibitors to factor replacement therapy. The median (range) age of the group (*n*=43) was 10.0 (6.2–15.1) years. All boys with severe haemophilia, 4 with moderate and 2 with mild disease were on prophylaxis with factor replacement. All boys with an inhibitor had been on prophylaxis for ≥ 6 months. Annual Bleed Rate (ABR) and known joint arthropathy frequency was low. Median (range) HJHS for the whole group was 1 (0–12); only 1 child had X-ray evidence of joint arthropathy (see Table 2). Boys do not routinely receive X-ray or MRI investigation unless clinically indicated. The majority (72%) of the boys had a normal BMI (Table 3).

**Table 2:** Participant age and disease characteristics

Characteristics	Mild (*n*=5)	Moderate (*n*=5)	Severe (*n*=26)	Inhibitor (*n*=7) (all severe)	Total group (*n*=43)
Median age (years) (range)	12.6 (9.3–14.6)	9.1 (6.7–15.1)	10.3 (6.3–14.6)	8.6 (6.23–11.15)	10.0 (6.2–15.1)
**Diagnosis:**					
Haemophilia A	5	2	20	7	34
Haemophilia B	0	3	6	0	9
**Treatment:**					
Prophylaxis	2	4	26	n/a	32
On demand	3	1	0	0	4
Novel treatment	n/a	n/a	n/a	7	7
Number of children with bleeds in previous year	2	0	10	0	12
Median Annual Bleed Rate (ABR) (range)	0 (0–1)	n/a	0 (0–3)	n/a	0 (0–3)
Known joint arthropathy	0	0	0	1 (right knee)	1
Median HJHS score^a^ (range)	1 (0–1)	0 (0–2)	1 (0–12)	3 (0–8)	1 (0–12)

^a^Of 43 boys 16 had HJHS lower limb scores >1, of which, median HJHS score=2 (range 1–6)

**Table 3:** Participant anthropometric characteristics

Characteristics	Mild (*n*=5)	Moderate (*n*=5)	Severe (*n*=26)	Inhibitor (*n*=7)	Total group (*n*=43)
Median height (cm) (range)	156.4 (123.7–169)	131.1 (115–163.1)	142.7 (112.2–175)	141.6 (113.9–150)	142.3 (112.2–175)
Median weight (kg) (range)	52.4 (23–59.9)	29 (20.7–44.8)	34.1 (19-71.9)	32.8 (17.4–46.8)	33.5 (17.4–71.9)
Median BMI (kg/m^2^) (range)	19.2 (15–5)	16.9 (13.5–18.4)	16.3 (13.2–23.5)	16.3 (13.4–21.6)	16.8 (13.2–25.8)
**BMI centiles:**					
95th and above (obese)	1	0	1	1	3
85th–94th (overweight)	0	0	3	1	4
5th–84th centile (normal)	4	4	19	4	31
Below 5th (underweight)	0	1	3	1	5

### Feasibility outcomes

NIHR and feasibility metric [3, 14] outcomes were applied to the study and indicate the protocol is feasible (see Table 4).

**Table 4:** Adapted from Learmonth and Motl (2018) [14]

Metric and reason	Example of feasibility objectives	Result of feasibility outcome in this study
**Process**—assesses the feasibility of processes that are key to future studies	Determine recruitment rates; e.g. the response of participants to recruitment strategies, proportion who remain interested/eligible after information and screening. Determine ease of randomisation	Consent rate 70% (*n*=46/66) Progression to recruitment after screening 93% (*n*=43/46) Easy due to random design, so all components completed by all participants.
**Resource**—assesses time and resource problems that could occur in future studies	Estimate retention of participants in the study e.g. number of participants completing all aspects of the study, number and reason for attrition. Demonstrate appropriate eligibility criteria e.g. are inclusion/exclusion criteria too relaxed/stringent Estimate barriers/refusals to participation Estimate access to/cost of equipment, space, personnel time Clinician training needs and competence.	100% of participants completed exercise testing on the day of clinic review. 95% (41/43) of the boys returned the accelerometer (1 lost, 1 unaccounted for).] 84% (36/43 boys) were fully compliant with the entire protocol, wearing the accelerometer long enough for valid data analysis of at least 4 days of 9 h [15]. Exclusion criteria included a bleed in the previous 6 weeks. 8 boys were not eligible for approach at clinic review due to a recent bleed. This was not considered too stringent and enhanced safety of exercise testing. Reasons such as additional time commitment/burden of exercise testing on the day of routine clinic review was too great for 17% (11/66 declined). Costs covered by study funds, space available for all exercise testing. The approximate cost of equipment: HR monitor with chest strap and Polar watch £260; Reebok step £80; stop watch £10; tape measure £4. All test procedures are free. A corridor of 12m is required for 10-m ISWT. Study completed within proposed timeframe; target of 40 boys in 1 year (43 in 10 months achieved). Initial training and supervision required for administering physiotherapy assessment protocol.
**Management**—assess potential human and data management problems.	Estimate equipment usage e.g. ease of availability, equipment malfunction. Staff annual leave/sickness Determine processing time for data collection e.g. time to mail data collection materials Data collection and analysis – software accessibility	Established that accelerometry and heart rate recording equipment would require updating prior to any further studies. 3% (2/66) of interested eligible boys not recruited on days when they could have participated due to researcher unavailability. Needs were met by at least 8 accelerometers, required for turnaround time of 1 week accelerometry-wear, postage time back to Centre, and subsequent downloading of data onto specialist software, off-site. Access to SPSS and ActiGraph Firmware was required, available in this research setting.
**Scientific**—assess the safety, burden, data collection and response to the study	Estimate challenges perceived/experienced by study personnel e.g. skills required to use assessment protocol Safety to boys of outcome measures Burden of research Determine appropriateness of target group Determine acceptability to participants e.g. participants views on outcomes.	Training and practice required to implement outcome measures. Greater subjectivity in use of the iSTEP compared with the 10-m ISWT, with regard to stopping boys. Strength of tester versus participant, in myometry testing – difficulty resisting muscle activity in older, stronger boys, but intra-rater reliability should remain stable. Challenges faced by boys in adverse conditions for exercise testing i.e. very high summer temperatures. No serious adverse events or reactions, but caution required and does not mean that there are no risks i.e. falls and trips. Significant amount of additional activity, alongside routine clinic review. Potential delayed onset muscle soreness in the days following exercise testing. Pain experienced during testing did not cause undue discomfort or distress. Time commitment burden of at least 1.5 hours in addition to routine clinic visit, plus 1 week of accelerometry-wear, measuring usual physical activity. Difficulties for some boys performing the iSTEP: reduced co-ordination, related to younger children in the cohort. Provided awareness of this. Boys were asked for verbal feedback on preferred exercise test and reasons. Both exercise tests were generally acceptable to participants.

### Process

Recruitment of 43 boys was achieved in 10 months, exceeding the proposed recruitment of 40 boys in 1 year. The consent rate was 70% (46/66). Progression to recruitment after screening was 93% (43/46): 3 boys were excluded due to bleed or injury present on the day.

### Resource

Data collection for all the patient-centred outcome measures increased the standard clinic review time by at least one and a half hours (HJHS is already part of clinic review for all except those with mild disease). Accelerometry data collection required a further commitment of 7 days’ wear plus postal return. Myometry, HJHS, and accelerometry have all been used in CYPwH ([5, 11, 15] respectively), but the practicalities of implementing them together as part of a study protocol in clinic has not been investigated. All boys completed the study protocol on the day of clinic review with full data collection achieved and no participant attrition. All boys received an accelerometer on the day of their review; a high return rate of 95% (41/43; 1 lost, 1 unaccounted for) was achieved. 84% (36/43) were able to wear the accelerometer for valid data analysis, of *at least* 4 days of 9 h [15]. 8% (5/66 boys) of boys approached could not take part due to a recent known bleed or injury. 17% of families approached (11/66) declined participation (mean age 11.4 vs total study cohort mean age 10.3). Reasons given were time constraints, younger siblings accompanying, and 4 of these boys (aged 11, 13, 14 and 15) declined themselves, not wanting to exercise. In this setting, 1 year of 30 h per week researcher time and equipment costs were covered by study funds; space was available to carry out all exercise testing. In terms of ease of use for the tester, both tests required training and practice to implement the outcome measures according to standardised instructions. The tester felt that more practice was necessary to implement the iSTEP compared to the 10-m ISWT.

### Management

Eight accelerometers and ActiGraph firmware were required for daily activity data collection, downloading and calculation; plus access to the statistical package, SPSS, for data management. This feasibility study established that, for any further studies seeking to investigate cardiovascular outcomes/maximal exercise testing, HR recording equipment should have the capacity to capture peak HR *during* testing. 3% of interested eligible boys were not recruited on days when they could have participated due to researcher unavailability.

### Scientific

There were no SAR/SAEs in any boys. There were some safety concerns highlighted by the study: During the 10-m ISWT, one boy slipped and fell onto his elbow and another described ‘different’ knee joint pain, both when decelerating around the cones. Both boys were stopped from completing testing*.* Pain resolved for both boys within minutes, with no issues reported the next morning when telephoned. A further boy was stopped, as his shoe fell off. During iSTEP testing, 3 boys were stopped by the tester for safety reasons; one boy lost co-ordination and was at risk of tripping/falling; the second boy tripped and slightly turned his ankle, but did not report pain as a result. A third boy was stopped because he had tripped twice, had reduced co-ordination and the tester acted cautiously as he had an inhibitor. As a measure of acceptability and safety, boys were asked to rate any type of pain experienced in any part of their body using the WBFPRS, during performance testing: Pain was reported by 19/43 (44%) boys during the iSTEP; 17/43 (40%) experienced no pain at all and 7/43 (16%) reported pain either immediately after-testing and/or at 3 min recovery. All boys continued/were allowed to continue in spite of pain, as reports fitted a clinical picture of step-up exercise-induced discomfort i.e. bilateral thigh pain and/or posterior calf pain. The maximum score given for pain during or after the iSTEP, using the WBFPRS, was 4/10 from several participants for reasons such as ‘dry throat’, ‘hard work for the legs’, ‘tired chest’.

iSTEP pain scores were comparable to pain scores for the 10-m ISWT, where 18/43 (42%) boys reported pain immediately post-testing and 3/43 (7%) scored pain only at 3 min recovery (pain scores were not taken *during* the 10-m ISWT). 22/43 (51%) scored no pain at all. The maximum pain score given by 2 different boys was 6/10, both for ‘tummy’ pain, which could have been cramp or a ‘stitch’. Pain during or after the iSTEP and 10-m ISWT did not have any apparent link to test order. See Table 5.

**Table 5:** Summary of pain and participant characteristics

	Pain experienced during or after performance testing			
	iSTEP		10-m ISWT	
	Yes	No	Yes	No
**Number of boys**	26	17	21	22
**Test order:**				
**iSTEP 1st**	15	8	13	10
**10-m ISWT 1st**	11	9	8	12
**Disease severity:**				
**Mild**	3	2	3	2
**Moderate**	2	3	3	2
**Severe**	15	11	12	14
**Inhibitor**	6	1	3	4
**Median age in years (range)**	9.9 (6.2–15)	10.2 (6.4–13.9)	10.2 (6.3–15)	9.9 (6.2–14.6)

One parent was telephoned after the study for a separate reason but reported that her son had experienced bilateral posterior thigh pain for 2 days following participation. She had given him extra factor. This was likely to have been normal delayed onset muscle soreness.A clear explanation to all boys was provided during consenting, regarding post-exercise muscle soreness being a normal response to exercise.

After participation, all boys were asked which performance test they preferred and why. 11 preferred the iSTEP, 29 the 10-m ISWT and 3 had no preference. The majority who preferred the 10-m ISWT did so because they ‘like running’. The majority who preferred the iSTEP found it ‘easier’ than the 10-m ISWT and liked that they could aim for level 5. 12 of the boys who preferred the 10-m ISWT displayed co-ordination difficulties on the iSTEP, recorded in tester notes.

## Discussion

This study has demonstrated the feasibility, safety and acceptability of using a protocol of selected outcome measures and performance tests in this cohort of boys with haemophilia. No SAR/SAEs occurred during testing and recruitment to the study was completed within the proposed timeframe. The 65% recruitment rate, with 95% retention rate at the end of participation at 1 week, suggests families would commit to these additional measures included in either clinical care or research. Only 17% of all those approached in this study declined participation. This is in comparison to feasibility studies in other child and adolescent populations [29–31] reporting lower recruitment and retention rates; for example, 25% and 77% respectively, and high percentages declining, e.g. 44.6%. The positive rates in our study may reflect the engagement families have with the haemophilia service, their motivation to contribute to research, the relatively low burden of research, and demonstrates the overall protocol acceptability and utility for future studies. The sample may have been self-selecting; those already engaged with exercise could be more likely to consent. The older mean age of the group declining participation may reflect evidence that sedentary behaviours of young people increase with age [32] or other factors associated with teenage years, including independence from parents or burden of research already experienced by this small cohort.

Myometry testing, the iSTEP and 10-m ISWT were all safe assessment tools in this study cohort. However, one boy fell during the 10-m ISWT and whilst low risk for an unaffected population, a fall in CYPwH could result in a more serious event. Whilst there were no falls during iSTEP testing, tripping was a reason for stopping three boys. Neither performance test in this study stands out as being more or less safe than the other. All exercise tests pose risks and supervision is essential for all tests regardless of patient population.

Understanding reports of pain as part of the bigger clinical picture in haemophilia is important. Pain can be an indication of bleeding and should always be fully assessed [33]. During performance testing, if a child had pain the tester used subjective and objective information in clinical decision making to stop testing or not. This was deemed more appropriate than employing set pain score thresholds to determine stopping. Unaffected boys performing the iSTEP similarly reported pain in 19/22 cases, scoring up to 7/10 on the visual analogue scale (Additional file 2). This supported our approach to allow normal, exercise-related pain during performance testing in boys with haemophilia, yet proceed with caution in the event of non-exercise related symptoms.

Boys’ preference for 10-m ISWT versus iSTEP was 29 versus 11 (3 liked both tests equally), with a zero rate of withdrawal from the study, suggesting acceptability of both performance tests. The 25-level 10-m ISWT entailed familiar actions of walking and running and therefore accommodated all abilities, but took longer than the iSTEP to administer in this setting (45 versus 20 min). The iSTEP requires repetitive, co-ordinated stepping and is capped at 5 levels, totalling 10 min. Time, space and cost are important feasibility considerations [6]. The 10-m ISWT requires 10m of dedicated space, preferably pre-marked on the floor, which may not be available in many settings. The iSTEP test was performed in a small space consistent with any clinic room, using inexpensive, portable equipment. Both tests have standardised audio instructions. The panel of measures included in this study protocol produced useful and discriminatory data in boys with haemophilia and will be reported in future publications.

### Study strengths and limitations

This study did not include boys with clinical evidence of synovitis, and only one boy with arthropathy participated. As such, no conclusions regarding the feasibility, acceptability or safety for boys with joint damage can be made.

Patient-reported quality of life measures were not included in the protocol, and further research would benefit from selecting only one functional performance measure and substituting the other with patient-reported aspects of measuring health, in line with International Classification of Functioning, Disease and Health (ICF) (WHO) [34].

Burden of research could be explored in future studies by collecting data on parent/carer and CYP opinion about time commitment and whether this affects participation.

This study used strict exclusion criteria compared to a similar study investigating exercise testing in 13 boys with haemophilia [35], one of whom had an ankle joint bleed the day after ergometry testing. He had experienced a same-joint bleed in the week preceding testing; those authors concluded a bleed-free interval of at least a week could be considered. The 6-week bleed-free period used in this study is considered more appropriate; to enhance safety and minimise risk of research-induced SAE/SARs.

### Key lessons learned and outcomes from this feasibility study

Updated heart rate recording technology is required so that heart rate can be monitored continuously throughout performance testing rather than just at set points during testing. In this way, more data can be accurately gathered i.e. peak heart rate.

iSTEP testing is feasible, safe and acceptable, which justifies further research to establish psychometric properties prior to its use as a validated, reliable outcome measure. This has enabled securing more funding, and such testing is underway.

The 25-level 10-m ISWT can be considered a safe, validated, reliable outcome measure for use in CYPwH, previously not tested in this cohort. However, it is without associated normal data for the UK, which reduces its utility as an outcome measure. Future research should address this.

## Conclusion

This study has demonstrated the feasibility, safety and acceptability of this study protocol in boys aged 6–15 years old with haemophilia A or B of any severity. It can therefore be used in larger clinical intervention trials, once psychometric properties of the iSTEP are established, and this is currently underway. The panel of tests was well tolerated in this cohort and risks associated with slipping, tripping or falling during high intensity performance testing were minimised by strict eligibility criteria and precautions applied during testing. No SAR/SAEs occurred during the protocol, which could be incorporated during routine clinic appointments or research, provided eligibility and precautions are considered. Both functional performance measures appeared safe and acceptable in this cohort, although the 10-m ISWT was the test preferred by most boys. The iSTEP required less time and space to administer and has associated normal data. Clinical space, boys’ ability and time may aid clinician choice.

## Supplementary Information

**Additional File 1.** Consort Checklist of information to include when reporting a pilot or feasibility trial.

**Additional file 2.** Title of spreadsheet: iSTEP data in typically developing children and young people.

## Data Availability

Data generated or analysed during this study are included in this published article. Any data not discussed will be made available in future publications. One dataset collected during iSTEP development [22] (iSTEP pain scores in unaffected peers) was used during the current study and is attached as Additional file 2.
