# Supplementary material for: Feasibility, safety and acceptability of select outcome measures in a physiotherapy study protocol for boys with haemophilia
Source: Pilot Feasibility Stud. 2021 May 6;7:105. doi: 10.1186/s40814-021-00831-1 (PMC8099992; doi:10.1186/s40814-021-00831-1)
Supplement: Supplementary file 2 — Additional file 2. Title of spreadsheet: iSTEP data in typically developing children and young people. [file 40814_2021_831_MOESM2_ESM.pdf]

Decimal age	Weight (kg)	Height (cm)	Sex	Height of step (cm)	Test date	SaO2 (%) rest	SaO2 (%) minimum	%change in SpO2	SaO2 (%) 1 min recovery
unavailable data*			M	15	18/08/2011	97	94	3	97
			M	20	18/08/2011	100	97	3	98
6.5	17.3	110.5	F	15	02/02/2012	94	93	1	97
6	23.5	123	M	15	02/02/2012	97	92	5	97
7	24.2	120.5	M	15	02/02/2012	95	91	4	94
6.3	18.8	115	F	15	02/02/2012	99	94	5	99
7	26.4	128.5	M	15	02/02/2012	98	93	5	99
7.3	24.9	121.5	M	15	02/02/2012	97	96	1	98
7.1	22.1	121.5	F	15	02/02/2012	95	94	1	98
7.4	24.2	128	F	15	02/02/2012	99	93	6	98
6.4	20.3	116	M	15	02/02/2012	96	95	1	93
7.3	23.5	122	F	15	02/02/2012	95	91	4	99
7.2	26.4	129.5	F	15	02/02/2012	99	95	4	98
6.6	23.3	118	F	15	02/02/2012	99	92	7	98
9	27.3	138	M	15	29/11/2011	96	96	0	97
8.6	32.1	138	F	15	29/11/2011	97	94	3	97
8.5	25.7	133.5	F	15	29/11/2011	98	93	5	98
8.8	38.1	131.5	F	15	29/11/2011	98	93	5	96
8.5	30.3	131.5	F	15	29/11/2011	96	92	4	97
8.9	30.6	133	F	15	29/11/2011	95	93	2	98
9.1	37.8	143.5	F	15	29/11/2011	96	94	2	98
8.9	32.05	135.5	F	15	29/11/2011	97	94	3	97
9.9	27.5	135.5	M	15	05/07/2012	98	97	1	98
9.9	28.8	138	F	15	05/07/2012	100	95	5	98
10.5	33.8	138	F	15	05/07/2012	100	98	2	98
10.5	29.7	138.5	M	15	05/07/2012	98	93	5	98
10.8	36	144	M	15	05/07/2012	98	97	1	97
10	33	138	F	15	05/07/2012	98	97	1	98
10.3	42.5	144.5	M	15	05/07/2012	99	96	3	99
10.7	56	152	F	15	05/07/2012	98	95	11	97
10.8	33.3	137.5	F	15	05/07/2012	99	96	3	98
10.8	46.7	151.5	F	15	05/07/2012	98	96	2	99
10.2	30.3	130	F	15	05/07/2012	99	98	1	98
13	63.8	165.8	M	20	13/02/2013	98	96	2	96
12.1	48.5	145.6	F	20	13/02/2013	98	95	3	98
12.1	43.5	145.2	F	20	13/02/2013	99	95	4	98
11.5	44.5	152	F	20	13/02/2013	100	98	2	97
12.4	38.3	144.5	M	20	13/02/2013	98	96	2	97
13.4	45.6	159.5	M	20	13/02/2013	98	94	4	98
12.5	55	153.5	F	20	13/02/2013	98	97	1	98
11.7	50	148	M	20	13/02/2013	98	97	1	98
12.3	79.3	168	F	20	13/02/2013	100	97	3	94
12	60.6	164	M	20	13/02/2013	97	96	1	96
13.4	51.8	171	F	20	13/02/2013	100	98	2	98
11.6	34.1	143	M	20	13/02/2013	98	97	1	98

14.6	62.2	176.5	M	20	10/02/2015	100	97	3	100
13.5	63.1	159.5	M	20	10/02/2015	100	97	3	97
13.7	33	156.5	M	20	10/02/2015	98	97	1	100
15	57.9	171	M	20	10/02/2015	98	97	1	99
16.3	70.9	176	M	20	10/02/2015	98	98	0	99
14.7	86.5	183	M	20	10/02/2015	99	98	1	98
Key:									
Unavailable data									

HR rest	HR_{max}	% HR_{max} achieved	HR 1 min recovery	OMNI rest	OMNI 1	OMNI 2	OMNI 3	OMNI 4	OMNI 5	OMNI 1 min recovery	VAS before
108	175	83.73	116	0	2	2	3	3	3	1	
89	179	86.89	150	0	1	2	3	4	4		
114	163	80.41	139	0	4	6	9		10	8	0.1
113	176	89.57	124	0	1	3	4	2	5	5	0.1
104	174	88.87	143	0	2	3	5	7	8	6	0
82	168	83.50	144	0	1	1	3	8	7	6	0
83	157	81.10	125	0	0	0	1	0	0	0	0
118	174	89.19	147	0	0	1	0		2	0	1
94	173	87.42	142	0	1	3	4	6	9	7	0.2
110	182	92.95	165	0	3	4	5	6	8	7	0
106	125	62.59		0	6	7	7			2	0.1
90	168	85.50	110	0	2	6	4	4	10	7	0.1
107	187	96.59	180	0	4	6	8	8	9	6	0
102	177	89.98	138	0	1	2	4	6	10	7	0
101	172	89.26	131	0	2	4	4	6	8	6	0.2
106	199	105.91	132	0	2	4	6	6	8	4	0.3
80	191	98.30	100	0	4	6	4	8	10	10	0.6
107	182	100.05	127	0	2	6	8	9	10	10	0.3
96	179	94.36	108	0	0	2	2	4	8	8	0.4
97	171	90.29	99	2	0	10	8	9	10	6	2.3
129	186	102.09	165	0	0	2	2	2	2	2	0.1
100	204	108.54	160	0	2	2	2	2	2	2	0
73	183	95.06	82	1	2	4	6	7	9	7	0.4
91	181	94.67	133	0	2	0	4	8	10	0	0
87	207	111.17	153	0	1	1	2	2	3	3	0
94	178	93.54	132	1	3	2	4	7	7	2	0.1
100	203	110.33	100	0	1	1	2	2	3	1	0.3
84	186	99.47	113	1	1	2	2	1	3	2	0.3
54	181	101.97	91	0	1	2	2	1	1	0	0
92	169	103.05	150	0	1	2	3	6	8	2	0
70	173	92.66	113	0	0	1	1	2	2	0	0
91	198	114.25	128	0	2	2	3	4	7	4	0
97	187	98.58	144	0	2	4	7	8	9	7	1.1
104	183	117.16	146	0	2	3	3.5	5	6	5.5	0
106	205	119.53	154	0	4	6	8	10	10	7	1
80	182	103.12	118	2	2	3	6			6	1
80	189	107.69	150	0	2	4	6	7	8	8	0
101	187	102.92	145	2	2	4	4	6	8	6	0
70	186	106.65	132	0	1	3	5	6	8	6	0
79	205	124.24	150	0	3	5	6	8	9	7	2
128	205	120.59	180	0	1	4	6	8		5	0
136	204	144.99	164	0	3	5	7	9	10	10	0
82	183	114.81	114	2	2	3	4	4	4	1	1
85	177	105.23	145	2	6	8				9	5
94	188	101.13	153	2	2	6	8	10		6	2

125	170	107.73	132	0	1	2	3	3	4	2	0
104	181	115.36	146	0	2	3	5	8		4	0
102	189	101.07	131	1	3	4	5	7	7	5	0
68	187	115.36	128	1	2	3	3	4	6	3	0
85	176	118.04	124	0	1	2	3	4	4	2	0
83	161	120.60	120	0	0	0	1	1	2	1	0

VAS 1 min recovery	Test complete (Y/N)	Test terminated by investigator (Y/N)	Reasons for termination			
	Y	N	N/A			
	Y	N	N/A			
0.2	Y	N	N/A			
0.2	Y	N	N/A			
0.5	Y	N	N/A			
1.8	Y	N	N/A			
0	Y	N	N/A			
0.2	Y	N	N/A			
3.8	Y	N	N/A			
3.8	Y	N	N/A			
0.1	N	Y	1, 4, 9			
1.2	Y	N	N/A			
5.5	Y	N	N/A			
4.9	Y	N	N/A			
3.3	Y	N	N/A			
2.9	Y	N	N/A			
9.2	Y	N	N/A			
4.3	Y	N	N/A			
4.7	Y	N	N/A			
7.1	Y	N	N/A			
1	Y	N	N/A			
1.7	Y	N	N/A			
4.8	Y	N	N/A			
2.3	Y	N	N/A			
0	Y	N	N/A			
1.1	Y	N	N/A			
0	Y	N	N/A	*Had been running around playgro		
1.3	Y	N	N/A			
0.1	Y	N	N/A			
1.7	Y	N	N/A	Sats monitor not working very wel		
0	Y	N	N/A			
3.4	Y	N	N/A			
4.3	Y	N	N/A			
5	Y	N	N/A			
9	Y	N	N/A			
7	N	Y	wheeze			
4	Y	N	N/A			
5	Y	N	N/A			
7	Y	N	N/A			
5	Y	N	N/A			
7	N	N	SOBOE++			
9	Y	N	N/A			
1	Y	N	N/A			
8	N	N	tired and loss of co-ordination			
5	N	N	LL fatigue and L calf pain			

3	Y					
7	N	N	SOB and slight wheeze			
5	Y					
4	Y					
0	Y					
1	Y					

ound for 1/2 hour prior to test		
l		
